# Effect of COVID-19 on the relationship between Euro/USD exchange rate and oil price

**DOI:** 10.1016/j.mex.2021.101262

**Published:** 2021-02-07

**Authors:** Neluka Devpura

**Affiliations:** Department of Statistics, Faculty of Applied Sciences, University of Sri Jayewardenepura, Nugegoda, Sri Lanka

**Keywords:** Predictability, COVID-19, Time-varying

## Abstract

We investigate the relationship between the Euro-United States Dollar (Euro/USD) exchange rate and oil futures price using intra-day data. The dataset is on hourly basis from 01/07/2019 to 30/11/2020 and 17-hour per day, from 01:00am to 17:00pm. By employing a predictive regression model, we observe oil price has influenced Euro/USD exchange rate but the evidence is very limited. Further, when we control for the effect of COVID-19, this relationship vanishes. Overall, COVID-19 shows some effect on the exchange rate during March 2020.

• There is no predictive ability of oil price on Euro/USD exchange rate after controlling for COVID-19.

## Graphical abstract

**Figure:**
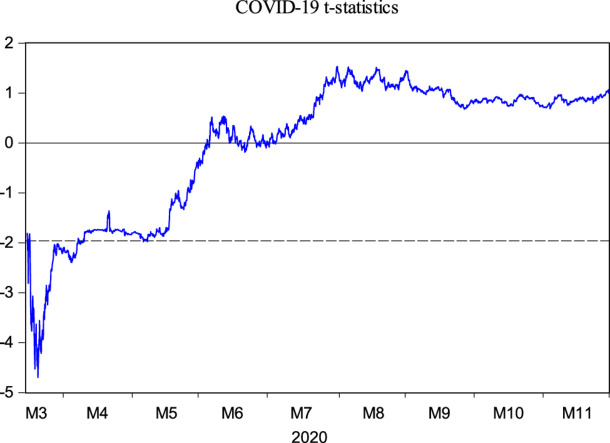

Specifications table

**Table:** 

Subject Area	*Economics and Finance*
More specific subject area	*Time Series*
Method name	*Predictive Regression Models*
Name and reference of original method	*Westerlund, J., & Narayan, P.K., 2012. Does the choice of estimator matter when forecasting returns? Journal of Banking and Finance, 36, 2632–2640.* *Westerlund, J., & Narayan, P.K., 2015. Testing for predictability in conditionally heteroskedastic stock returns. Journal of Financial Econometrics, 13, 342–375.* *Devpura, N. (2020). Can Oil Price Predict Japanese Yen? Asian Economics Letters Forthcoming.*
Resource availability	*–*

## Introduction

The COVID-19 pandemic has slowed down global economic progress (see [34], [35], [36]; Sha & Sharma, 2020; Sharma and Sha, 2020). In this paper, we propose the hypothesis that COVID-19 has influenced the relationship between oil prices and exchange rates (Euro/USD). The motivation for this comes from several papers that show how COVID-19 has impacted both financial and macroeconomic relations. In this literature, Iyke [17] and Narayan, Gong and Ahmed [31] find that COVID-19 is a predictor for exchange rates and stock returns, respectively.^1^ Narayan [25], [26], [27] shows how bubble activity has changed in exchange rates due to COVID-19; how exchange rate resilience to shocks have changed due to COVID-19; and how the oil market has changed as a result of COVID-19. In addition, Narayan et al. [30] show that exchange rate depreciation boosted stock returns in the COVID-19 period.^2^ Other studies offer equally important insights: Appiah-Otoo et al. [4], for instance, reveal using Chinese data that exchange rate significantly decreases domestic credit during the pandemic; Salisu and Sikiru [41] find that during the COVID-19 period, uncertainty of the pandemic is a factor for Asia Pacific Islamic stock returns; and Qin et al. [37] show that the pandemic has a negative effect on the oil price.

In this literature, no one has considered whether the ability of the oil price to predict exchange rates has changed. Considering the recent findings in the literature on how COVID-19 has been instrumental in changing the behavior of economic and financial variables, we were inspired to investigate the relationship between the exchange rate and oil futures prices (West Texas Intermediate (WTI) futures price) amidst the COVID-19 pandemic.^3^

The method we adopt to test our hypothesis is the predictive regression model proposed by Westerlund and Narayan [47], [48]. The appealing aspect of this model is that it handles the persistency, endogeneity, heteroskedasticity present in the variables. Hence, it has been extensively used in the literature to identify predictability of the various asset classes; such as stock returns [5], [10], [28], [29], [41], oil prices [8], exchange rates [39], and inflations [43].

We find limited evidence of time-varying predictive ability of oil in explaining the Euro/USD rate. And, once the COVID-19 predictor is introduced, oil becomes an ineffective predictor of the exchange rate. This paper contributes to the COVID-19 literature as identified above.

In Section II, we discuss the method we use, section III reveals the results from the regression models, and the final section provides the conclusions from this paper.

## Method

In this section, we discuss the methodology and data we adopted to test our hypothesis. Our sample consists of high frequency data: we have hourly data from 01/07/2019 to 30/11/2020. The data are 17-hour per day, from 01:00am to 17:00pm. The selected variables are exchange rate (ER) Euro to US Dollar (Euro/USD) and the oil price (OIL), which is the WTI 1-month oil futures price. We follow the regression model proposed by Westerlund and Narayan [47], [48]. We start by calculating the response variable, ER, as the natural log percentage returns as follows:

(1) $$ER=\;\left(\text{ln}\left(ER_{t}\right)-\text{ln}\left(ER_{t-1}\right)\right)*100$$

Devpura [8] uses the model below which controls for econometric issues of persistency, endogeneity and heteroskedasticity. Thus, heteroskedasticity is controlled by dividing each variable by its standard deviation. Here, we employ two regression models to find predictive ability of the OIL price variable. The first model is stated below and it is estimated by ordinary least squares method:

(2) $$ER_{t}=\beta_{0}+\beta_{1}OIL_{t-1}+\beta_{2}\left(OIL_{t}-OIL_{t-1}\right)+\varepsilon_{t}$$

In addition to the variables defined earlier, $\varepsilon_{t}$ is the disturbance term. In this regression, $\left(OIL_{t}-OIL_{t-1}\right)$ controls for persistency and endogeneity of the predictor variable, OIL. The null hypothesis that $\beta_{1}=0\;$tests no predictability.

In the second model, we augment Eq. (2) to control for the COVID-19 pandemic by including a dummy variable, COVID. In order to define the COVID variable, we consider the date on which COVID-19 was declared officially as a pandemic by World Health Organization.^4^ Hence, the date is 11th March 2020. Therefore, we use 0′s to represent pre-COVID-19 period and 1′s to represent COVID-19. Thus, pre-COVID sample is from 01/07/2019 to 11/03/2020 and COVID-19 sample consists from 12/03/2020 to 30/11/2020. See the expanded model as follows;

(3) $$ER_{t}=\beta_{0}+\beta_{1}OIL_{t-1}+\beta_{2}\left(OIL_{t}-OIL_{t-1}\right)+\beta_{3}COVID+\varepsilon_{t}$$

We run these Eqs. (2) and (3) to obtain the time-varying coefficients to examine the time-varying predictability of oil. For this, we use an expanding window approach. In this method, we use the first 50% of the sample as in-sample and run the regression model, estimating the parameters. Then, we increase the sample by one hour and re-run the regression model to obtain time-varying estimates. In this way, we continue recursively by expanding the window until the entire sample is used.

## Discussion of results

We now discuss some preliminary results followed by the main regression results. Table 1 reports the descriptive measures of raw data for the full sample (01/07/2019 to 30/11/2020) and the two sub-samples: namely, the pre-COVID-19 (01/07/2019 to 11/03/2020) sample and the COVID-19 (12/03/2020 to 30/11/2020) sample. For the Euro/USD rate, the mean (US$1.14) is highest in the COVID-19 sample. When we consider the volatility in terms of standard deviation, again the highest rate is found in the COVID-19 sample. Another feature is that the asymmetry is measured by skewness and it is positive for the full sample; however, in both the pre-COVID-19 and the COVID-19 samples, it is negative. When we look at the variable, *OIL*, the mean value is highest (US$55.60) in the pre-COVID-19 sample and lowest (US$35.27) in the COVID-19 sample. We notice the minimum *OIL* price is reported as negative US$7.65 and it is in the COVID-19 sample. The standard deviation is US$8.53 for COVID-19 sample and it is larger than the pre-COVID-19 sample. Also, the skewness measure is negative in all the three samples, indicating that the distribution is negatively skewed. When we use the Jarque-Bera test for normality, both Euro/USD and *OIL* reject the null of normality for each and every sample. Finally, we have the number of observations in the samples and we can claim that the pre-COVID and the COVID-19 samples are roughly the same at 3111 and 3196 observations, respectively.

**Table 1:** Descriptive Statistics This table shows the descriptive statistics for the variables: currency pair Euro/USD and West Texas Intermediate Oil futures prices (*OIL*). The second, third and fourth columns report the results for the full sample (01/07/2019 to 30/11/2020), Pre-COVID-19 sample (01/07/2019 to 11/03/2020) and COIVD-19 sample (12/03/2020 to 30/11/2020), respectively. The descriptive measures are Mean, Max (Maximum), Min (Minimum). Std. Dev. (Standard Deviation), Skew (skewness), Kurtosis, JB (Jarque-Bera) test for null of Normality, the corresponding probability for JB test and finally, it shows the number of observations for each sample.

	Full Sample		Pre-COVID-19 Sample		COVID-19 Sample	
Description	Euro/USD	*OIL*	Euro/USD	*OIL*	Euro/USD	*OIL*
Mean	1.13	45.30	1.11	55.60	1.14	35.27
Max	1.20	64.57	1.15	64.57	1.20	46.00
Min	1.07	−7.65	1.08	28.43	1.07	−7.65
Std. Dev.	0.03	12.26	0.01	4.56	0.04	8.53
Skew	0.70	−0.71	−0.10	−2.14	−0.31	−1.39
Kurtosis	2.14	2.97	3.51	11.50	1.51	4.38
JB	708.06	524.11	38.39	11732.31	347.57	1280.87
Probability	0.00	0.00	0.00	0.00	0.00	0.00
Observations	6307	6307	3111	3111	3196	3196

**Fig. 1:**
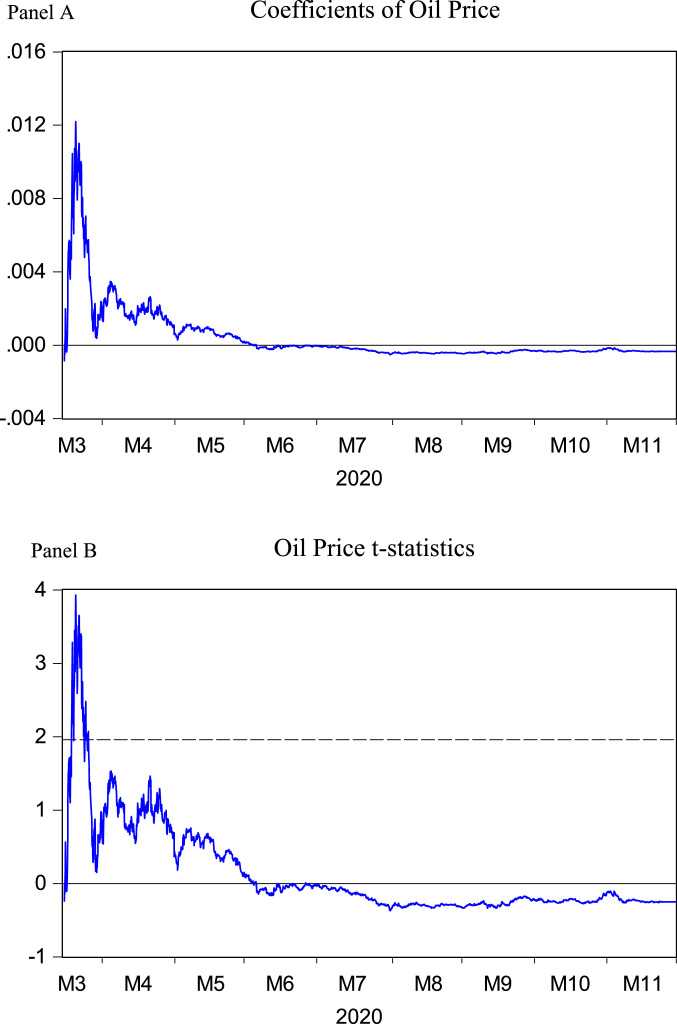
Time-varying results for oil price This figure depicts the regression results for the model, $ER_{t}=\beta_{0}+\beta_{1}OIL_{t-1}+\beta_{2}\left(OIL_{t}-OIL_{t-1}\right)+\varepsilon_{t}$. Here, $ER_{t}$ is the exchange rate of Euro/USD in natural log percentage return form, $OIL_{t}$ is the WTI 1-month oil futures price, and $\varepsilon_{t}$ is the disturbance term. Using the method of recursive window, the time-varying $\beta_{1}$ coefficients are estimated. For this, the data are divided into two sets. The first half of the data are used as an in-sample values and with these data we get the first coefficient for the parameter $\beta_{1}.$ Since, we employ recursive (expanding) window method, in the next step, we increase the sample by one-hour and we produce the second estimated ${\hat{\beta }}_{1}$. This is repeated until we consume the last observation of the data. We show the results in two panels, where Panel (A) indicates ${\hat{\beta }}_{1}$ coefficients and Panel (B) shows its’ time-varying t-statistics. The solid horizontal line cuts at 0 and the dotted line in Panel (B) at ± 1.96 which the significance of the ${\hat{\beta }}_{1}$ at 5% significance level.

We plot the time-varying results using time-series plots. Fig. 1 illustrates the time-varying coefficients (Panel A) and t-statistics (Panel B) for the oil price that we obtained from Eq. (2). We observe in the month of March 2020 some significant t-statistics that lie beyond 1.96 which is the 5% significance level. However, we do not have evidence for time-varying significance. Since, early July 2020 to the end of sample, we observe negative coefficients.

**Fig. 2:**
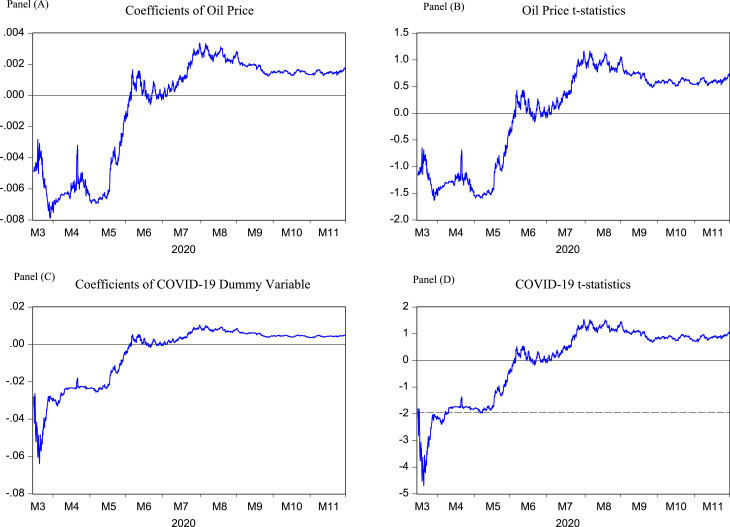
Time-varying results controlling for COVID-19 This figure illustrates the results from the model $ER_{t}=\beta_{0}+\beta_{1}OIL_{t-1}+\beta_{2}\left(OIL_{t}-OIL_{t-1}\right)+\beta_{3}COVID+\varepsilon_{t}$ Here, $ER_{t}$ is the exchange rate Euro/USD in natural log percentage return form, $OIL_{t}$ is the WTI 1-month oil futures price, COVID is a dummy variable with two categories pre-COVID is 0 and COVID-19 represent 1′s, and $\varepsilon_{t}$ is the disturbance term. Using the method of recursive window, the time-varying $\beta_{1}$ coefficients are estimated. For this, the data are divided into two sets. The first half of the data are used as an in-sample values and with these data we get the first coefficient for the parameter $\beta^{\prime }$s. We employ recursive (expanding) window method. We show the results in four panels, where Panel (A) indicates ${\hat{\beta }}_{1}$ coefficients and Panel (B) shows its’ time-varying t-statistics, Panel (c) illustrates ${\hat{\beta }}_{3}\;$and the relevant t-statistics are shown in Panel (D). The solid horizontal line cuts at 0 and the dotted line in Panel (D) is at ± 1.96 which is the significance of the ${\hat{\beta }}_{3}$ at 5% significance level.

Finally, we have the results from Eq. (3) plotted in Fig. 2. Panels A and B illustrate the coefficients of OIL and its corresponding *t*-statistics. When we control for COVID-19, we do not find any significant t-statistics for the oil variable. However, Panels C and D illustrate the time-varying coefficients and t-statistics for the COVID dummy. In Panel D, the dotted line is drawn at −1.96, which is the 5% level of significance. We notice that the COVID dummy variable is significant in the month of March 2020. Therefore, we find limited evidence of the effect of COVID-19 on the Euro/USD exchange rate.

## Conclusion

In this paper, using 17-hour data, from 1:00am to 5:00pm, over the period of 01/07/2019 to 30/11/2020, we investigate the relationship between the Euro/USD exchange rate and oil price. We find limited evidence of a time-varying predictability relationship between the variables. Our study contributes to the growing literature on COVID-19 and its effects on the financial and economic system by considering the oil price and exchange rate nexus during the pandemic.

COVID-19 pandemic is continuing and future research are likely to predict different asset prices in light of COVID-19 because new research (see for instance, [6], [16], [22], [24], [30], [32], [44], [49], [51] and [18]) has shown that COVID-19 has influenced the entire financial and economic system.

## Declaration of Competing Interest

The authors declare that they have no known competing financial interests or personal relationships that could have appeared to influence the work reported in this paper.

## Footnotes

1 The literature on COVID-19 has grown significantly and continues to progress. It is therefore impossible to cite all papers. Here, we cite those papers closest to our work in our opinion. For studies on the stock market and COVID-19 relationship, see for instance Prabheesh [33] for Indian stock market and foreign portfolio investments during COVID-19; Gil-Alana and Claudio-Quiroga [13] for COVID-19 impact on the Asian stock markets; Yan and Qian [51] for the impact of COVID-19 on the Chinese Stock Market; Liu et al. [22] for impact of COVID-19 on crude oil and stock markets in the US; Xu [50] for stock returns and COVID-19 relation in the US and Canada; He et al. [15] for COVID-19 impact on Chinese sectorial stock returns; Sergi et al. [42] for COVID-19 and stock market return relation of 76 countries; So et al. [45] for COVID-19 and Hong Kong stock returns; Ashraf [1] for stock market reactions to COVID-19 of 43 countries; Contessi and De Pace [7] for instability and crashes of 18 stock markets due to COVID-19; and Salisu and Akanni [40] for COVID-19 and stock returns of OECD countries. Haroon and Rizvi [14] find that COVID-19 cases are associated with the liquidity of financial markets. Interested readers can refer to Wei at al. [46] for a study on exchange rate and COVID-19.

2 One of the most influential strands of the COVID-19 literature relates to the energy sector; see, for instance, Devpura and Narayan [9], who find evidence of oil price volatility increasing in the COVID-19 period. Ertuğrul et al. [11] find evidence of a high volatility pattern of the Turkish diesel market during COVID-19. Polemis and Soursou [38] find that the pandemic influenced stock returns of the Greek energy firms. Akhtaruzzaman et al. [3] disclose COVID-19 as a moderator to the oil price shock. Gharib et al. [12] find contagion effect of bubbles in oil and gold markets during the COVID-19 pandemic. Amar et al. [2] show spillovers of commodity and stock prices in oil producing and consuming countries during the COVID-19 period.

3 See for instance Jung et al. [19] for oil and USD-Canada exchange rate; Krugman, [20] for oil shocks and exchange rate, Liu, Tan, and Wang [21] commodity prices and exchange rates etc.

4 Liu, Choo and Lee [23] investigated on pandemic announcement and find negative shock on the global stock market.
